# Comprehensive relaxometric analysis of Fe(iii) coordination polymer nanoparticles for *T*_1_-MRI: unravelling the impact of coating on contrast enhancement†

**DOI:** 10.1039/d5na00250h

**Published:** 2025-05-09

**Authors:** Marco Ricci, Fabio Carniato, Alessia Corrado, Giuseppe Ferrauto, Enza Di Gregorio, Giovanni Battista Giovenzana, Mauro Botta

**Affiliations:** a Dipartimento di Scienze e Innovazione Tecnologica, Università del Piemonte Orientale Viale T. Michel 11 Alessandria 15121 Italy mauro.botta@uniupo.it; b Magnetic Resonance Platform (PRISMA-UPO), Università del Piemonte Orientale Viale Teresa Michel 11 15121 Alessandria Italy; c Department of Molecular Biotechnology and Health Sciences, University of Torino 10126 Torino Italy giuseppe.ferrauto@unito.it; d Dipartimento di Scienze del Farmaco, Università del Piemonte Orientale Largo Donegani 2/3 28100 Novara Italy; e Magnetic Resonance Platform (PRISMA-UPO), Università del Piemonte Orientale Via Bovio 6 28100 Novara Italy

## Abstract

Coordination polymer-based systems, particularly Fe(iii)-based polymers, are attracting increasing interest due to their well-controlled morphology, biocompatibility, and versatile surface functionalization. With five unpaired electrons, Fe(iii) offers a promising and safer alternative to Gd(iii) for MRI applications. While some studies have investigated low molecular weight Fe(iii) chelates for MRI, the exploration of Fe(iii)-based nanosystems as *T*_1_ MRI probes remains limited. This study focuses on the synthesis of Fe(iii)/gallic acid nanoparticles functionalized with a low molecular weight polyethylene glycol (PEG) shell, designed to enhance the second-sphere water interaction and improve *r*_1_ relaxivity at clinical magnetic fields. The ^1^H NMR relaxometric properties of these nanoparticles were systematically analyzed as a function of proton Larmor frequencies and temperature, and their performance was compared with a similar system stabilized by polyvinylpyrrolidone (PVP). We aimed to determine the frequency dependence of relaxivity in Fe(iii)-based coordination polymers, and to assess the impact of coating modifications on their MRI contrast efficacy. This knowledge is crucial for the rational design of improved Fe(iii)-based nanoprobes, allowing for optimized performance in future MRI applications.

## Abstract 2

Gallic acid-based Fe(iii) nanoparticles, coated with PEG, exhibit remarkable efficacy as *T*_1_ MRI contrast agents thanks to an improved second sphere hydration.

## Graphical abstract

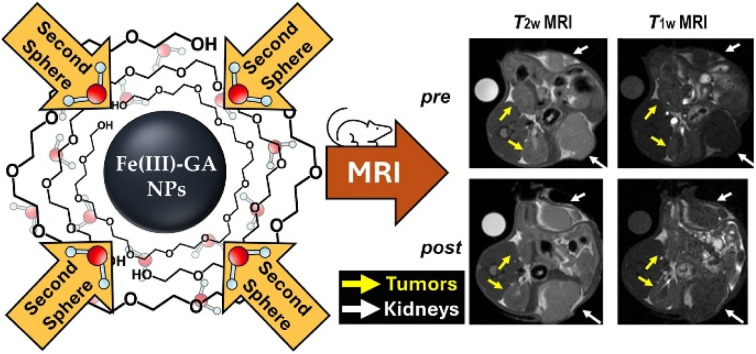

## 1. Introduction

In recent years, a wide variety of inorganic, organic, and hybrid nanoparticles composed of materials such as silica, carbon, oxides, liposomes, and nanogels have been developed and evaluated as potential *T*_1_ and/or *T*_2_ MRI probes.^1–10^ These efforts aim to provide viable alternatives to the Gd(iii)-based paramagnetic complexes currently used in clinical practice.^11–13^ Increasing attention has been directed toward coordination polymer-based systems, which have demonstrated significant promise for nanomedicine applications.^14^ These systems stand out due to their precisely controlled morphology, extensive compositional versatility, excellent biocompatibility, and the ease of surface functionalization. In this context, Fe(iii)-based coordination polymers hold great potential as promising alternatives to Gd(iii)-based MRI probes.^15^ High-spin Fe(iii) possesses five unpaired electrons, resulting in a magnetic moment (*ca.* 5.9 B.M.) comparable to that of Mn(ii) complexes, which have long been investigated as substitutes for Gd(iii).^16^ Moreover, Fe(iii) offers a superior safety profile. As an essential element for life, Fe(iii) is naturally abundant in the human body, with an endogenous presence of approximately 3–5 grams, further supporting its biocompatibility and potential for biomedical applications. While numerous studies in the literature highlight the use of low-molecular-weight Fe(iii) chelates for MRI diagnostic applications,^17–23^ the exploration of Fe(iii)-based nanosystems as potential *T*_1_ MRI probes remains relatively limited. In 2015, Z. Wang *et al.*^15^ published a groundbreaking study introducing ultrasmall nanoparticles based on a Fe(iii) coordination polymer, constructed with gallic acid units and surface-stabilized by polyvinylpyrrolidone (PVP) chains, as a promising system for *T*_1_-MRI applications. However, the investigation of their magnetic properties, crucial for their application as MRI diagnostic probes, remains quite unexplored. From a relaxometric perspective, this system exhibited a longitudinal relaxivity (*r*_1_ = 1.4 mM^−1^ s^−1^ at 1.5 T and 310 K) at clinical magnetic fields strengths (1.5 T) comparable to those of low molecular weight Fe(iii) complexes. However, its relaxivity was significantly lower than that of commercially available Gd(iii) chelates, which typically achieve values around 3.5 mM^−1^ s^−1^ under similar conditions.^11^ Nevertheless, this study demonstrated that these particles could effectively accumulate within tumour tissues, making them suitable for both diagnostic purposes and photothermal therapy. More recently, additional formulations stabilized with different coatings or chemically modified on the surface through covalent bonding with polyethylene glycol chains, have been developed, incorporating gallic acid in combination with drugs or targeting vectors.^24–26^

Studies on other types of nanoparticles, such as Gd(iii) fluorides,^27^ have shown that optimizing the surface coating layer can significantly enhance relaxometric properties. In particular, hydrophilic coatings are known to attract water molecules to the nanoparticle surface, facilitating stronger magnetic interactions between the exposed paramagnetic centres and the protons of nearby water molecules, thereby improving overall relaxivity.

Driven by these considerations, this study pursued two primary objectives:

(a) Comprehensive magnetic characterization: we performed a thorough magnetic characterization in aqueous solution utilizing state-of-the-art instrumentation. This included a detailed investigation of the ^1^H NMR relaxometric behavior as a function of proton Larmor frequency and temperature. To the best of our knowledge, the only macromolecular system based on Fe(iii) for which a frequency-dependent relaxometric study has been conducted and quantitatively analyzed consists of supramolecular adducts of human albumin with [Fe(EDTA)]^−^ derivatives.^28^

(b) Relaxivity enhancement through coating modification: we aimed to enhance the nanoparticles' relaxivity by substituting the PVP coating with PEG during synthesis, thereby maximizing water–Fe(iii) ion interactions due to PEG's superior hydrogen bonding capabilities with solvent molecules. Additionally, PEG offers a significant advantage over PVP in tumor-targeting applications by extending circulation time in the bloodstream and enhancing the passive accumulation of nanoparticles in tumors *via* the enhanced permeability and retention (EPR) effect. Furthermore, PEG can be functionalized with specific ligands for active targeting, further increasing the precision of drug delivery to tumor cells.^29,30^

Beyond relaxometric studies, we conducted a comprehensive morphological and spectroscopic analysis, comparing our PEG-functionalized nanoparticles with a PVP-stabilized system.^15^ This comparison allowed us to assess the impact of the coating on the morphology and surface properties of the resulting nanosystems. Finally, we evaluated the biocompatibility of our formulation through both *in vitro* and *in vivo* studies and investigated its potential as a photothermal agent for cancer therapy.

## 2. Experimental

### 2.1 Materials

All chemicals were purchased from Sigma-Aldrich Co. and used without further purification.

#### 2.1.1 Synthesis of Fe(iii)-GA NPs

Fe(iii)–GA NPs were synthesized adapting a procedure reported in literature.^15^ The Fe(iii)–GA NPs were synthesized with a molar ratio of coating : Fe(iii) : gallic acid of 0.01 : 0.12 : 0.06 in the starting reactant solution. Two different types of NPs, differentiated by the chemical nature of the coating used in the reaction mixture (polyvinylpyrrolidone PVP MW = 8000 Da and polyethylene glycol PEG MW = 4000 Da) were synthesized.

#### 2.1.2 PEG-based NPs (PEG/Fe(iii)–GA)

**Fig. 1:**
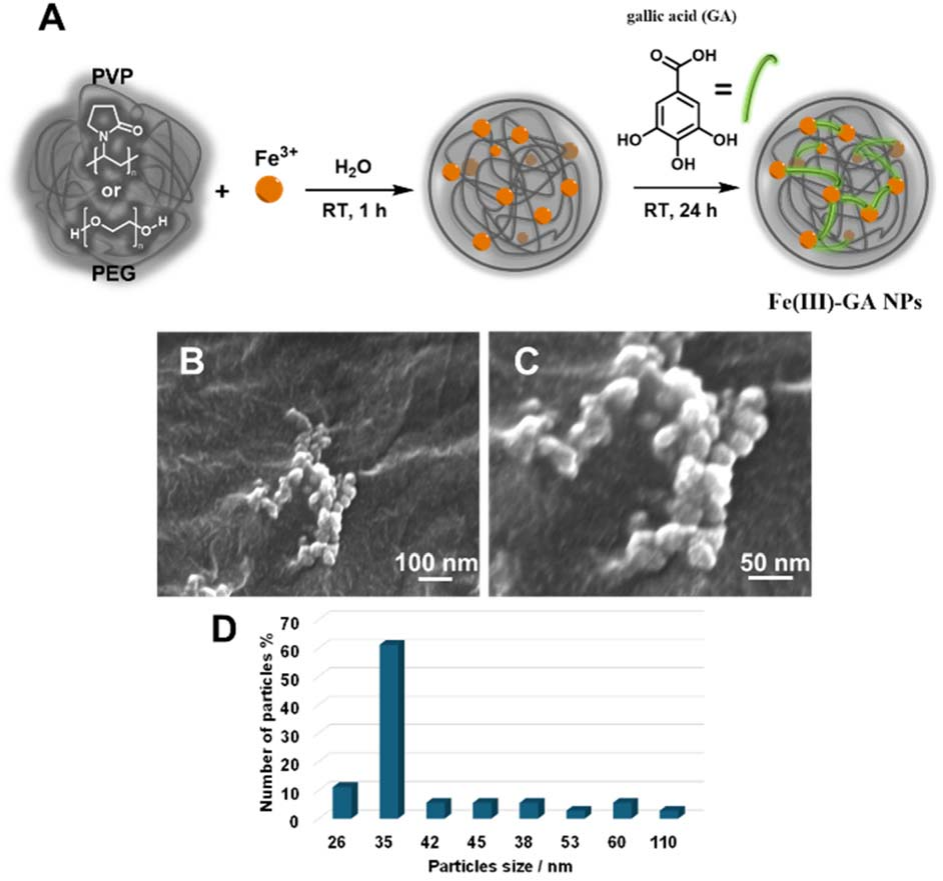
(A) Schematic view of the synthesis of PEG/Fe(iii)–GA and PVP/Fe(iii)–GA nanoparticles. SEM micrographs of PEG/Fe(iii)–GA NPs at low (B) and high magnifications (C). (D) Particles size distribution.

PEG (40.0 mg) was dissolved in milliQ water (8.8 mL) under stirring (Fig. 1). Then, an aqueous solution of FeCl_3_·6H_2_O (33.3 mg of iron chloride in 0.2 mL of milliQ water) was added and left for 1 hour under vigorous stirring. The solution appears as pale yellow (Fig. S1†). Finally, a gallic acid (GA) aqueous solution (10 mg in 1 mL of milliQ water) was added to the reaction mixture and stirred overnight. Immediately, the solution turned dark green, due to the complexation of the Fe(iii) ions by GA ligand (Fig. S1†). The final suspension with a pH of 2.2 was then dialyzed (MWCO = 14 000 dalton) in deionized water for 24 h. During the dialysis, a gradual change in the colour of the suspension is observed (from green to dark red), associated with a change of pH from 2.2 to neutrality. The same synthetic approach was conducted for the PVP-based NPs (PVP/Fe(iii)–GA), used as reference, by simply substituting PEG with PVP (80 mg).

### 2.2 Characterization techniques

Infrared (IR) spectra were collected in the range 4000–400 cm^−1^ with a Bruker Equinox 55 spectrometer: all measures were collected by diluting the samples in KBr matrix.

Scanning electron microscopy measurements were performed using a ZEISS GeminiSEM 360, a high-resolution field emission scanning electron microscope. After deposition of 1 mg of samples on the sample holder, NPs were coated with a layer of few nm of platinum using the Emitech K575X metallizer, to enhance conductivity and minimize charging effects during the analysis.

The density of the NPs was measured using the Anton Paar Ultrapyc 5000 gas pycnometer at room temperature. The instrument was calibrated with standard reference materials to ensure accuracy. For the experiment, the sample was placed in a measurement cell, and the system was purged with argon to eliminate air. The gas flow was regulated to maintain consistent pressure and temperature conditions throughout the experiment.

Dynamic light scattering (DLS) and *ζ*-potential experiments were carried out at 298 K with a Malvern Zetasizer NanoZS operating in a particle size range from 0.6 nm to 6 μm and equipped with a He–Ne laser with *λ* = 633 nm.

UV-vis spectra of the nanoparticles suspensions were carried out by using a Lambda 900 UV-visible spectrometer (PerkinElmer, Waltham, Massachusetts, USA).

Elemental analyses were performed with an Ametek Spectro Genesis EOP Inductively Coupled Plasma Atomic Emission Spectrometer (ICP-AES) (Kleve, Germany) equipped with a cross-flow nebulizer with simultaneous spectrum analysis in the 175–770 nm range. The suspensions were mineralized with concentrated HNO_3_ at 373 K for 24 h.

1/*T*_1_^1^H Nuclear Magnetic Relaxation Dispersion (NMRD) profiles were measured on a Fast-Field Cycling (FFC) Stelar SmarTracer Relaxometer over a wide range of applied magnetic field strengths, from 0.00024 to 0.25 T (0.01–10 MHz proton Larmor Frequencies). The relaxometer operates under computer control with an absolute uncertainty in 1/*T*_1_ of ±1%. Data in the 20–120 MHz frequency range were collected with a High Field Relaxometer (Stelar) with a HTS-110 3T Metrology Cryogen-free Superconducting Magnet. The temperature during the measurements was controlled trough a Stelar VTC-91 airflow heater equipped with a copper-constantan thermocouple (uncertainty of ±0.1% °C). The data were collected using the standard inversion recovery sequence (20 experiments, 2 scans) with a typical 90° pulse width of 3.5 μs, and the reproducibility of the data was within ±0.5%. The 1/*T*_2_ data were collected with a standard CPMG sequence, with a reproducibility of the data within ±1.0%. Additional points at 500 MHz were collected by using a Bruker AVANCE III 500 spectrometer equipped with a wide bore 11.7 tesla.

*In vitro* experiments were conducted by dissolving 90 mg of human serum (Seronorm™, LOT 1512606) in 1 mL of the suspension before the measurement.

The ^17^O NMR data were collected using a Bruker Avance III spectrometer (11.7 T) with a 5 mm probe under controlled temperature conditions. An aqueous solution of the NP was enriched to 2.0% of the ^17^O isotope (Cambridge Isotope). The transverse relaxation rates were determined from the signal width at half-height across a temperature range of 275–350 K.

### 2.3 Biological analyses

Cell toxicity (MTT and haemolysis): to evaluate the potential *in vivo* availability, the biocompatibility of NPs was assessed *in vitro* using the standard MTT assay and a haemolysis assay to determine their impact on cell viability and on Red Blood Cells, respectively. For MTT assay, TS/A murine breast cancer cells were used. TS/A murine breast cancer cells were derived at the University of Torino from a spontaneous mammary adenocarcinoma which arose in a retired breeder BALB/c female.^31^ They were grown in RPMI^1064^ supplemented with 10% (v/v) heat-inactivated fetal bovine serum (FBS), 2 mM glutamine, 100 U per mL penicillin and 100 mg per mL streptomycin (purchased from Lonza Sales AG, Verviers, Belgium). Cells were seeded in 75 cm^2^ flasks at density of *ca.* 2 × 10^4^ cells per cm^2^ in a humidified 5% CO_2_ incubator at 310 K. When cells reached confluence, they were detached by adding 1 mL of Trypsin–EDTA solution (0.25% (w/v) Trypsin 0.53 mM EDTA). Cells were negative for mycoplasma as tested by using MycoAlert™ Mycoplasma Detection Kit by Lonza (Lonza Sales AG, Verviers, Belgium).

For cytotoxicity MTT assay,^32^ TS/A cells were seeded into 96-well tissue culture plate (10^4^ cells for plate) 24 h before the experiment. Then, they were incubated with fresh complete RPMI medium in presence of NPs at variable concentrations (0 ÷ 1 mM of iron) for 24 h at 310 K. After the incubation time, medium was removed, cells washed and re-incubated in presence of fresh RMPI medium supplemented with 0.5 mg per mL MTT (Thiazolyl Blue Tetrazolium Bromide, Sigma-Aldrich) for 4 h in a humidified 5% CO_2_ incubator at 310 K. Then, MTT solution was removed, and plates were filled with DMSO (0.1 mL for plate) for ½ h at room temperature, under gentle agitation, for allowing solubilization of formazan crystals. The absorbance of the resulting-coloured solutions was quantified using a 96-multiwell iMark Bio-Rad microplate Reader (*λ* = 570 nm). The background provided by the presence of NPs in the cell medium is subtracted from the measured value. The percentage of viable cells was calculated based on control blank cells by using the following formula (1):

1 Viable cells % = (Abs_T_/Abs_ctrl_) × 100

where Abs_T_ is the mean absorbance of treated cells and Abs_ctrl_ is the mean absorbance of control untreated cells (after subtraction of absorption of empty plates as background). Cells experiments were repeated in quadruplicate and data reported as mean ± standard deviation. Blank was repeated 10 times.

Haemolysis assay was performed on red blood cells (RBCs) collected from the tail vein of 14–16-week-old male BALB/c mice weighing approximately 25 ± 3 g, using a 27-gauge syringe preloaded with heparin. Blood was diluted in fresh PBS and centrifuged at 2300 rpm for 8 minutes to pellet the cells. The RBCs were washed, recentrifuged, and then exposed to NPs at concentrations of iron ranging from 0.1 mM to 1 mM for 30 minutes at room temperature. Post-incubation, samples were centrifuged, and the supernatant was collected to measure the released haemoglobin using spectrophotometry at 413 nm (Soret's band) with a 6715 UV/vis Spectrophotometer (JEOL). RBCs incubated in fresh PBS served as controls. RBCs lysed using milliQ water were used as positive control, for calculating the total amount or released Hb. The percentage of haemolysis was calculated by using the following formula (2):

2 Haemolysis % = ((Abs_T_ − Abs_ctrl_)/(Abs_FR_)) × 100

where Abs_T_ is the mean absorbance (*λ* = 413 nm) of treated RBCs and Abs_ctrl_ is the mean absorbance of control RBCs treated with fresh PBS (negative control) and Abs_FR_ is the mean absorbance of RBCs totally lysed by osmotic shoch (incubation in milliQ water 1 : 6 v/v, osmolarity of *ca.* 50 mOsm L^−1^). Cells experiments were repeated in triplicate and data reported as mean ± standard deviation. Blank was repeated 5 times.

### 2.4 Magnetic resonance imaging

Magnetic resonance images (MRI) were acquired with a Bruker Avance 300 Spectrometer equipped with a 2.5 microimaging probe (*B*_0_ = 7.1 T). Phantoms were prepared by filling glass tubes with NPs at variable concentrations (0.02 ± 1 mM of iron). Preliminary scout images were acquired for organizing the geometry. Axial *T*_2_-weighted (*T*_2w_) images were acquired at 7.1 T using a Rapid Acquisition with Refocused Echoes (RARE) sequence (TR 4000 ms; TE effective 41 ms; RARE factor 128; slice thickness 1 mm; FOV 12 mm × 12 mm; acquisition matrix 128 × 128; spatial resolution 0.094 × 0.094 mm^2^; number of averages = 4). Axial *T*_1_-weighted (*T*_1w_) images were acquired using a standard multislice multiecho (MSME) sequence (TR 250 ms; TE 3.3 seconds; slice thickness = 1 mm; FOV 12 mm × 12 mm; acquisition matrix 128 × 128; spatial resolution 0.094 × 0.094 mm^2^; number of averages = 6). Regions of interest (ROIs) were manually drawn. *T*^enh%^_1_ were calculated as follows (3):

3 *T*^enh%^_1_ = ((SI − SI_0_)/SI_0_) × 100

where SI is the signal intensity of the specimen and SI_0_ the signal intensity of the reference (water) recorded using the proper *T*_1w_ sequence as above reported. Measurement of *T*_1_ was carried out by using a Saturation Recovery Spin Echo sequence (TE = 3.8 ms, 10 variable TR ranging from 50 to 5000 ms, FOV = 1 cm × 1 cm, slice thickness = 1 mm, matrix size 128 × 128). *T*_2_ values were measured by using a MSME sequence (TR = 2000 ms, 20 variable TE ranging from 11 to 500 ms, FOV = 1 cm × 1 cm, slice thickness = 1 mm, matrix size 128 × 128).

### 2.5 *In vitro* photothermal effect

The photothermal effect of the nanoparticles (NPs) was evaluated *in vitro* by monitoring the temperature of the samples under varying durations of light irradiation (up to a maximum of 1 hour). For this purpose, 0.5 mL of each sample was placed in a glass vial, which was then enclosed in heat-insulating polystyrene foam and positioned inside a thermally insulated polystyrene box. This setup effectively minimized heat exchange with the environment. A small opening at the top of the box allowed laser light to penetrate and directly irradiate the sample. Temperature measurements were carried out using a dual thermocouple system, which simultaneously recorded both the sample temperature and the ambient air temperature inside the box, ensuring accurate monitoring of thermal variations during irradiation. For excitation, a 1 cm collimated laser beam with *λ* = 680 nm was used (dB electronic instruments, Bresso (Mi)-Italy). The energy power was calculated using a Digital Handheld Optical Power and Energy Meter Console (Compact Power and Energy Meter Console) equipped with a Standard Photodiode Power Sensor, Si, (*λ* = 400–1100 nm, power range = 500 nW–500 mW) (ThorLabs, Incs). The energy power of the laser source was checked before any experiment. The irradiation density value is always constant at 0.48 W cm^−2^. Before the execution of the experiments, an equal volume of buffer was placed in the vials and subjected to the same excitation protocol. No change of temperature was measured upon 1 h of light irradiation. Firstly, the comparison of temperature enhancement for NPs and ctrl at the same concentration of iron (*i.e.* 5 mM) was carried out. Then, NPs were tested at variable concentration of iron (*i.e.* 5 mM, 3 mM, 1 mM, 0.5 mM and 0.1 mM). Each experiment was performed in triplicate and results reported as mean ± SD.

### 2.6 *In cellulo* photothermal effect

The efficacy of PTT was tested in TS/A cells. TS/A cells were seeded into 96-well tissue culture plate (10^4^ cells for plate) 24 h before the experiment. Then, they were incubated with fresh complete RPMI medium in presence of PEG/Fe(iii)–GA NPs at variable concentrations (0 ÷ 0.5 mM of iron) for 24 h at 310 K. After the incubation period, the medium was removed, and the cells were washed and re-incubated with fresh RPMI medium. Each plate was then exposed to laser irradiation for 20 minutes following the previously described protocol. After irradiation, the plates were placed in an incubator for 2 hours, after which cell viability was assessed using the MTT assay, as previously described.

### 2.7 Animals and *in vivo* MRI

As an animal model of breast cancer, 8–10-week-old female BALB/c mice, weight of 24 ± 3 g (Charles River Laboratories, Calco, Italy), were used for the subcutaneous injection of breast cancer cells.

Mice were kept in standard housing with standard rodent chow, water available ad libitum, and a 12 h light/dark cycle.

Experiments were performed according to the Amsterdam Protocol on Animal Protection, in conformity with institutional guidelines that are in compliance with national laws (D. L. vo 116/92, D. L. vo 26/2014 and following additions) and international laws and policies (2010/63/EU, EEC Council Directive 86/609, OJL 358, Dec 1987, NIH Guide for the Care and Use of Laboratory Animals, U.S. National Research Council, 1996). This study was carried out in the framework of a protocol approved by the Italian Ministry of Health (authorization number808/2017-PR).

For tumour-model preparation, mice were anesthetized *via* an intramuscular injection of tiletamine/zolazepam (Zoletil 100; Virbac, Milan, Italy) 20 mg per kg plus xylazine (Rompun; Bayer, Milan, Italy) 5 mg kg^−1^ using a 27-G syringe.

For the preparation of syngeneic murine models of cancer, about 4 × 10^5^ TS/A cells were suspended in 0.1 mL of phosphate buffer and subcutaneously injected into both the flanks of each mouse. Mice were used after 2 weeks from implantation when the tumour reached a volume of *ca.* 300 mm^3^.

Each mouse was implanted with two tumours to increase the number of analysed samples. Tumour growth was monitored through manual measurements using a calliper, and MRI scans were performed two weeks after implantation (*N* = 3 mice). For MRI experiments, anaesthesia was administered *via* intramuscular injection of tiletamine/zolazepam (Zoletil 100; Virbac, Milan, Italy) 20 mg per kg plus xylazine (Rompun; Bayer, Milan, Italy) 5 mg kg^−1^. For MRI, Fe(iii) nanoparticles were intraperitoneally administered at a dose of 0.05 mmol (Fe) per kg b.w. (b.w. = body weight).

Magnetic resonance images (MRI) were acquired with a Bruker Avance 300 Spectrometer equipped with a 2.5 microimaging probe (*B*_0_ = 7.1 T) before and after (up to 24 h) the intraperitoneal administration of Fe(iii) nanoparticles ([Fe] = 0.05 mmol per kg b.w., volume 0.1 mL). Axial *T*_2_-weighted (*T*_2w_) images were acquired using a Rapid Acquisition with Refocused Echoes (RARE) sequence (TR 4000 ms; TE effective 41 ms; RARE factor 128; slice thickness 1 mm; FOV 12 mm × 12 mm; acquisition matrix 128 × 128; spatial resolution 0.094 × 0.094 mm^2^; number of averages = 4). Axial *T*_1_-weighted (*T*_1w_) images were acquired using a standard multislice multiecho (MSME) sequence (TR 200 ms; TE 3.3 seconds; slice thickness = 1 mm; FOV 12 mm × 12 mm; acquisition matrix 128 × 128; spatial resolution 0.094 × 0.094 mm^2^; number of averages = 6). Regions of interest (ROIs) were manually drawn. *T*^enh%^_1_ was calculated with eqn (3).

## 3. Results and discussion

### 3.1 Synthesis and chemical characterization

The synthesis of PEG/Fe(iii)–GA nanoparticles is achieved by reacting iron chloride with gallic acid in a 2 : 1 molar ratio in an aqueous solution at room temperature, using low molecular weight polyethylene glycol (PEG, MW = 4000 g mol^−1^) as a stabilizing agent (Fig. 1A and S1†). This PEG-coating strategy builds upon a previously established method for a similar nanosystem that utilized polyvinylpyrrolidone (PVP) as a stabilizing shell.^15^ For comparison, PVP-stabilized Fe(iii)–GA (PVP/Fe(iii)–GA) nanoparticles were also synthesized as a reference material (Fig. 1A).

The Fe(iii) ion content in the nanoparticles, determined by ICP-MS analysis, was found to be 0.05 mmol g^−1^ for PEG/Fe(iii)–GA and 0.12 mmol g^−1^ for PVP/Fe(iii)–GA. The nanoparticle density, comparable in both cases, ranged from 2.1 to 2.3 g cm^−3^ (Table 1).

**Table 1:** Morphological and chemical properties of PEG/Fe(iii)–GA and PVP/Fe(iii)–GA nanoparticles

	Hydrodynamic diameter (nm)	*ζ*-Potential (mV)	Density (g cm^−3^)	Fe(iii) content (mmol g^−1^)
PEG/Fe(iii)–GA	108 ± 18	−24.2 ± 4.3	2.10 ± 0.12	0.05 ± 0.01
PVP/Fe(iii)–GA	6 ± 1	−8.7 ± 5.3	2.32 ± 0.03	0.12 ± 0.02

**Fig. 2:**
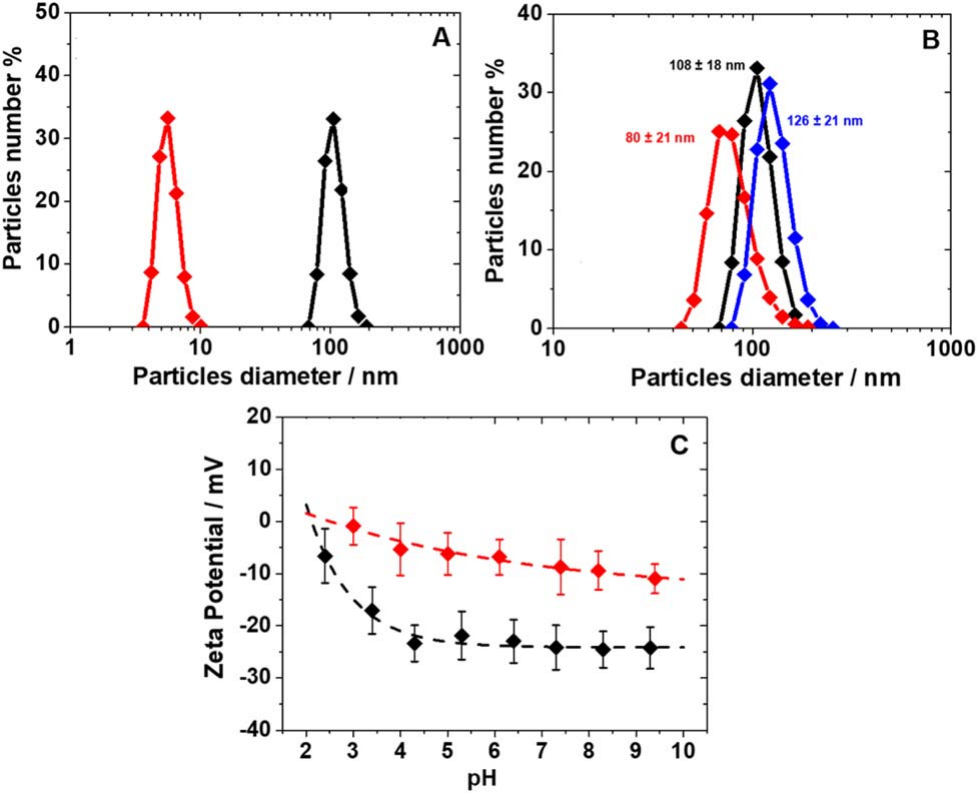
(A) DLS profiles of PEG (black, [Fe^3+^] = 0.17 mM) and PVP/Fe(iii)–GA NPs (red, [Fe^3+^] = 0.06 mM) in water at pH 7.4; (B) DLS of PEG/Fe(iii)–GA NPs at pH 3.4 (red), 7.4 (black) and 8.8 (blue); (C) *ζ*-potential data for PEG (black) and PVP/Fe(iii)–GA (red) as a function of pH.

The morphology of PEG/Fe(iii)–GA nanoparticles was examined using scanning electron microscopy (SEM). Micrographs captured at both low and high magnifications revealed spheroidal nanoparticles with diameters below 120 nm (Fig. 1B and C). Size distribution analysis of approximately 100 nanoparticles indicated an average particle size of around 35 nm (Fig. 1D). Notably, PEG/Fe(iii)–GA nanoparticles were larger than PVP/Fe(iii)–GA particles, which have been previously reported to be under 10 nm. This size difference may result from variations in the polymeric coating's ability to regulate particle growth during synthesis. The two formulations exhibit significant differences in particle size and surface charge distribution, even in aqueous solutions. As previously noted, PEG/Fe(iii)–GA has a hydrodynamic diameter of approximately 100 nm at neutral pH, which is nearly ten times larger than that of PVP/Fe(iii)–GA (Fig. 2A). Dynamic light scattering (DLS) analysis reveals that both formulations exhibit slight size variations with pH changes, with particle size distributions ranging from 80 to 130 nm under acidic (pH 3.4) and basic (pH 8.8) conditions (Fig. 2B). Both PEG/Fe(iii)–GA and PVP/Fe(iii)–GA aqueous suspensions remained stable at pH 7.4 and 298 K for up to two weeks, showing no signs of sedimentation or aggregation. This stability is primarily attributed to their negative surface charge, measured at −24.2 mV for PEG/Fe(iii)–GA and −8.7 mV for PVP/Fe(iii)–GA, as determined by *ζ*-potential analysis (Table 1). At pH values below 4, a progressive reduction in surface charge is observed for both formulations, likely due to the protonation of gallic acid carboxylate groups on the particle surface (Fig. 2C).

The presence of the PEG coating on the nanoparticle surface was confirmed using infrared (IR) spectroscopy. The IR spectrum of PEG/Fe(iii)–GA exhibited characteristic absorption bands in the 3000–2800 cm^−1^ range and around 1100 cm^−1^, corresponding to the C–H and C–O stretching modes typical of PEG, verifying the presence of the polymeric shell. Additionally, peaks observed in the 1700–1600 cm^−1^ region and at lower wavenumbers were attributed to the vibrational modes of the gallic acid units (Fig. S2†). Furthermore, the UV-visible spectrum of PEG/Fe(iii)–GA displayed a broad absorption band centred at 550 nm, attributed to d–d electronic transitions of Fe(iii).^15^ This spectral feature suggests a 1 : 3 stoichiometry of Fe(iii) to GA within the complex units, in agreement with previously reported data for similar nanosystems (Fig. S3†).^15^

### 3.2 Relaxometric analysis

The relaxometric properties of PEG/Fe(iii)–GA and PVP/Fe(iii)–GA nanoparticles were evaluated by analysing their proton longitudinal relaxivity (*r*_1_) across different magnetic fields (^1^H NMRD profiles) and temperatures. The parameter *r*_1_ represents the increase in the longitudinal relaxation rate of water protons in the presence of a 1 mM concentration of the paramagnetic metal ion.^33,34^ The *r*_1_ values of PVP/Fe(iii)–GA at 0.5, 1.5, and 3.0 T were measured in the range of 1.4–1.6 mM^−1^ s^−1^ at 298 K. Notably, PEG/Fe(iii)–GA exhibited a significant enhancement in *r*_1_, reaching 4.1 mM^−1^ s^−1^ at 3.0 T and 298 K, corresponding to a relaxivity of approximately 5800 mM^−1^ s^−1^ per particle, assuming each particle contains around 1400 Fe(iii) ions, an increase of approximately 150% compared to PVP/Fe(iii)–GA (Table 2). Furthermore, the *r*_2_/*r*_1_ ratios for both formulations remained close to 1 at clinical magnetic field strengths (1.5–3.0 T), indicating that these nanoparticles could serve as effective positive MRI contrast agents.^35^

**Table 2:** *r*_1_ and *r*_2_/*r*_1_ of PEG/Fe(iii)–GA and PVP/Fe(iii)–GA nanoparticles at different magnetic fields (298 K, pH = 7.4)

	0.5 T		1.5 T		3.0 T	
	*r*_1_/mM^−1^ s^−1^	*r*_2_/*r*_1_	*r*_1_/mM^−1^ s^−1^	*r*_2_/*r*_1_	*r*_1_/mM^−1^ s^−1^	*r*_2_/*r*_1_
PVP/Fe(iii)–GA	1.4	1.1	1.5	1.3	1.6	1.6
PEG/Fe(iii)–GA	2.8	1.2	3.3	1.2	4.1	1.2

For both samples, the *r*_1_ values measured at high magnetic fields were higher than those observed for non-hydrated Fe(iii) chelates (*e.g.*, [Fe(DTPA)]^2−^).^18^ This increase could initially be attributed to inner-sphere hydration of the Fe^3+^ ions embedded within the polymer matrix. However, considering the 1 : 3 stoichiometry of Fe(iii) to gallic acid in the complex units at neutral pH, this hypothesis warrants reconsideration. An alternative explanation involves the contribution of a second-sphere water layer, where water molecules interact *via* hydrogen bonding with the polar groups of the chelates. To explore these aspects in greater detail, we collected ^1^H NMRD profiles and conducted high-resolution ^17^O NMR experiments, providing deeper insight into the hydration dynamics and relaxometric behaviour of these nanosystems.^35^

The *r*_1_ values were initially monitored as a function of pH at 298 K and 32 MHz to evaluate the chemical integrity of the nanoparticles and the stability of the suspensions under acidic and basic conditions. Both formulations exhibited constant *r*_1_ values across a broad pH range (5–10), indicating that the coordination sphere of the metal centres remained intact and that neither hydrolysis nor aggregation occurred within this pH range. Below pH 5, a gradual increase in relaxivity was observed. This phenomenon can be attributed to an increase in the hydration state of Fe^3+^ ions, likely resulting from a reduction in the ligand's coordination sites as previously reported in the literature (Fig. S4†).^15^ This observation is supported by variable-temperature transverse ^17^O NMR relaxivity (*r*_2_) measurements conducted at 11.7 T, pH 7.4, and 3.0. At physiological pH, both PEG/Fe(iii)–GA and PVP/Fe(iii)–GA exhibited negligible broadening of the ^17^O resonance compared to typical hydrated Fe(iii) probes.^18^ These data strongly suggest that neither nanosystems possesses inner-sphere water molecules (*q* ≥ 1) exchanging rapidly enough on the NMR timescale to significantly influence the broadening of the ^17^O water signal. However, tests conducted at acidic pH for both PVP/Fe(iii)–GA and PEG/Fe(iii)–GA revealed a distinct change in the *r*_2_ profile. The emergence of a peak with a maximum close to 310 K provides compelling evidence for a shift in the hydration state of the metal under mildly acidic conditions (Fig. S5†).

**Fig. 3:**
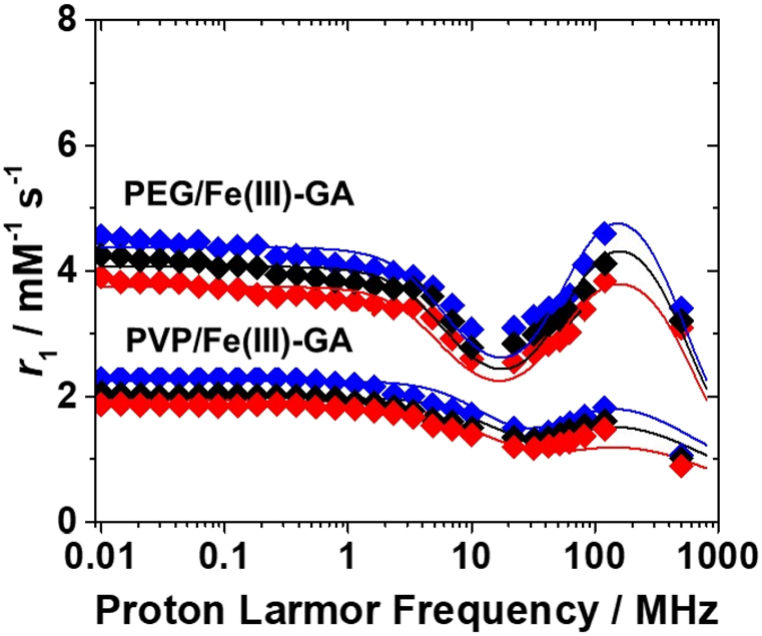
^1^H NMRD profiles of PEG/Fe(iii)–GA ([Fe^3+^] = 1.7 mM) and PVP/Fe(iii)–GA ([Fe^3+^] = 0.6 mM) at 283 (blue), 298 (black) and 310 K (red); pH = 7.4.

To better understand why PEG/Fe(iii)–GA nanoparticles exhibit superior relaxometric properties at clinical magnetic fields compared to PVP/Fe(iii)–GA, *r*_1_ values were measured across a broad range of magnetic fields (10 kHz to 500 MHz) and at different temperatures (283 K, 298 K, and 310 K) at pH 7.4 (Fig. 3). Since the Fe(iii)–gallic chelates in both nanoparticle formulations are not directly hydrated at neutral pH, their relaxivity is primarily governed by two mechanisms: (i) dipolar interactions with water molecules hydrogen-bonded to the polar groups of the chelate (second-sphere contribution, SS) and (ii) long-range interactions with bulk water molecules diffusing near the complexes (outer-sphere contribution, OS).^35^

For compounds whose relaxivity is mainly governed by the second-sphere mechanism, the longitudinal relaxivity is influenced by the number of second sphere water molecules (*q*^ss^), the residence time of water in the second sphere (*τ*^SS^_M_) and rotational correlation time (*τ*^SS^_R_), which describes the motion of the vector connecting the Fe(iii) ion to the SS water proton. Since second-sphere water molecules are not directly coordinated to the metal centre, they remain highly mobile and are only marginally affected by the rest of the nanoparticle. In contrast, both the number and the average lifetime of hydrogen-bonded water molecules have a significant impact on relaxivity.

The mathematical analysis of the ^1^H NMRD profiles,^36^ conducted using the Solomon–Bloembergen–Morgan (SBM)^37–39^ and Freed equations,^40,41^ accounts for both second- and outer-sphere contributions. An excellent fit was achieved by considering 0.1 and 0.75 second-sphere water molecules (*q*^SS^) for PVP/Fe(iii)–GA and PEG/Fe(iii)–GA, respectively, with a proton distance (*r*^SS^) of 3.1 from Fe(iii). The reorientational correlation time (*τ*^SS^_R_) was approximately 310 ps, while the water residence lifetime (*τ*^SS^_M_) was in the nanosecond range (Table 3). Other parameters, such as the minimum distance of outer-sphere water molecules (*a*) and the relative water and solute diffusion coefficient (*D*), were fixed to standard values.^18^

**Table 3:** Parameters (298 K) obtained by the global analysis of ^1^H NMRD profiles of PEG/Fe(iii)–GA and PVP/Fe(iii)–GA

Parameters	PVP/Fe(iii)–GA	PEG/Fe(iii)–GA
*Δ* ^2^/10^20^ s^−2^	4.7 ± 0.2	4.0 ± 0.2
*τ*_V_/ps	8.5 ± 0.2	7.0 ± 0.2
*τ* ^SS^_R_/ps	310 ± 2	308 ± 3
*τ* ^SS^_M_/ns	5.0 ± 0.1	7.1 ± 0.1
*q* ^SS^	0.10 ± 0.1	0.75 ± 0.02
*r* ^SS^	3.1^a^	3.1^a^
*a* ^SS^	3.5^a^	3.5^a^
^298^ *D*/m^2^ s^−1^	2.24 × 10^−10^^a^	2.24 × 10^−10^^a^

a Parameters fixed during the analysis.

The parameters related to electron spin relaxation, including the zero-field splitting mean squared energy (*Δ*^2^) and its correlation time (*τ*_V_), are consistent with values reported for numerous Fe(iii) chelates.^42^ In addition, given that the nanoparticle coating does not alter the structure of the coordination polymer, the electronic relaxation time of Fe(iii) ions are similar in both cases.

Due to the substantial chemical complexity of these paramagnetic nanoprobes, accurately fitting a single model to the ^1^H NMRD profiles presents a significant challenge. Furthermore, these profiles typically reflect the average contribution from all paramagnetic ions, assuming their equivalence. However, this assumption is not valid for these nanosystems. In these cases, only the Fe^3+^ ions exposed on the surface contribute significantly to the relaxometric properties. Nonetheless, though the system is complex, our conclusions are rigorously supported by relaxation data and appropriate paramagnetic relaxation equations.

Based on these results, it is evident that the increased *r*_1_ value for PEG/Fe(iii)–GA can be attributed to a higher average number of second-sphere water molecules. This likely stems from the more hydrophilic nature of the polymer coating on the particle surface. While the rotational correlation time (*τ*^SS^_R_), associated with the molecular tumbling of second-sphere water molecules, influences the shape of the ^1^H NMRD profile at high magnetic fields, its effect on relaxivity has a decidedly lower impact.

**Fig. 4:**
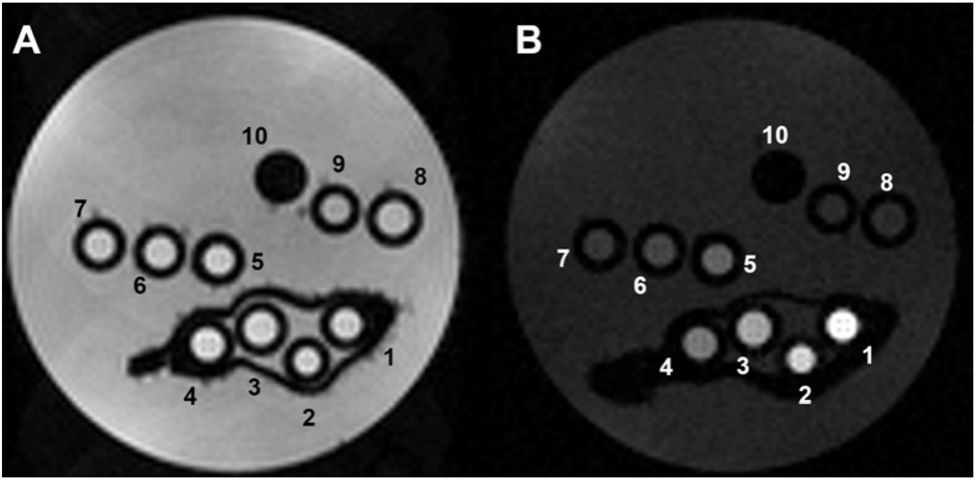
Representative *T*_2w_ (A) and *T*_1w_ (B) MR images of phantoms composed by glass capillaries filled with the Fe-NPs at variable iron concentration, as follows: (1) 1 mM, (2) 0.7 mM, (3) 0.5 mM, (4) 0.3 mM, (5) 0.2 mM, (6) 0.1 mM, (7) 0.05 mM, (8) 0.03 mM, (9) 0.02 mM and (10) empty reference capillary (*B*_0_ = 7.1 T).

Given the enhanced *r*_1_ values observed for PEG/Fe(iii)–GA at clinical magnetic field strengths compared to the PVP-coated sample, we further investigated the *T*_1_ and *T*_2_ MRI contrast properties of the pegylated nanoparticles. *T*_2_-weighted (*T*_2w_) and *T*_1_-weighted (*T*_1w_) MR images were acquired in phantoms composed of glass capillaries filled with PEG/Fe(iii)–GA at varying concentrations, at 7.1 T and 298 K (Fig. 4).

As expected, no *T*_2_ contrast was detectable (Fig. 4A). In contrast, a strong *T*_1_ contrast was observed, with hyperintensity in the *T*_1w_ MR image (Fig. 4B). The *T*_1_ enhancement (*T*^enh^_1_) reached significant values, up to 580 ± 50% for [Fe^3+^] = 1 mM (specimen 1) (Fig. S6†).

**Fig. 5:**
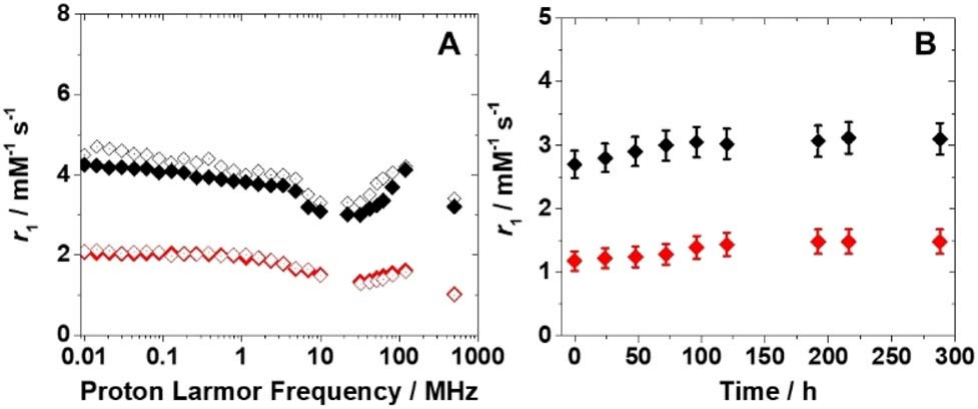
(A) ^1^H NMRD profiles at 298 K of PEG/Fe(iii)–GA ([Fe^3+^] = 1.7 mM) (black) and PVP/Fe(iii)–GA ([Fe^3+^] = 0.6 mM) (red) in water (full symbols) and in Seronorm™ solution (empty symbols). (B) Dependence of ^1^H *r*_1_ over time for PEG/Fe(iii)–GA (black) and PVP/Fe(iii)–GA (red) in Seronorm™ at 310 K.

Before considering potential *in vivo* applications, the stability of the aqueous suspensions and the chemical integrity of the nanoparticles were evaluated in a solution containing reconstituted human serum (Seronorm™). A total of 90 mg of lyophilized biological matrix was dissolved in 1 mL of an aqueous solution of PVP/Fe(iii)–GA and PEG/Fe(iii)–GA at pH 7.4. The longitudinal relaxivity of both formulations was measured across a range of fields (10 kHz–500 MHz) at 298 K. The relaxometric profiles were found to be comparable to those of pure aqueous suspensions (Fig. 5A), and the *r*_1_ values measured at 32 MHz and 310 K remained stable over twelve days, with only minor fluctuations (Fig. 5B). These results suggest that both nanosystems maintain stability in a biological medium, with no evidence of sedimentation or chemical degradation of the suspensions.

### 3.3 *In vitro* and *in vivo* analyses

**Fig. 6:**
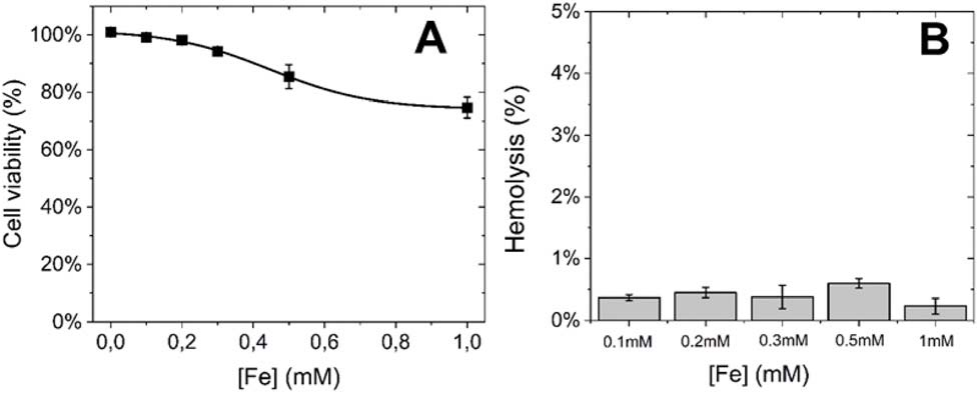
(A) Viability assessment of TS/A murine breast cancer cells after 24-hour incubation with varying concentrations of PEG/Fe(iii)–GA (incubation temperature = 310 K). (B) Percentage of RBC haemolysis following a 30-minute incubation with PEG/Fe(iii)–GA at different concentrations (incubation temperature = 298 K).

To assess the potential feasibility of *in vivo* administration, the biocompatibility of PEG/Fe(iii)–GA nanoparticles was evaluated *in vitro* using the standard MTT and haemolysis assays to determine their impact on cell viability and red blood cells (RBCs), respectively. For the MTT assay, TS/A murine breast cancer cells were incubated with nanoparticles at varying concentrations (0–1 mM of Fe(iii)) for 24 hours at 310 K. As shown in Fig. 6A, PEG/Fe(iii)–GA nanoparticles exhibited minimal toxicity, with cell viability remaining at approximately 75% after incubation with 1 mM Fe(iii) for 24 hours. Additionally, a haemolysis assay was conducted on RBCs collected from the tail vein of 14–16-week-old male BALB/c mice. The RBCs were exposed to PEG/Fe(iii)–GA nanoparticles at Fe(iii) concentrations ranging from 0.1 mM to 1 mM for 30 minutes at room temperature. After incubation, the samples were centrifuged, and the supernatant was collected to measure the released haemoglobin by monitoring absorption at 413 nm using UV-visible spectroscopy. As shown in Fig. 6B, no significant effect on RBCs was observed at any of the tested concentrations, with haemolysis remaining below 1% for all specimens. Both the MTT and haemolysis assays suggest that these nanoparticles are promising candidates for potential use in both cellular and *in vivo* applications.

**Fig. 7:**
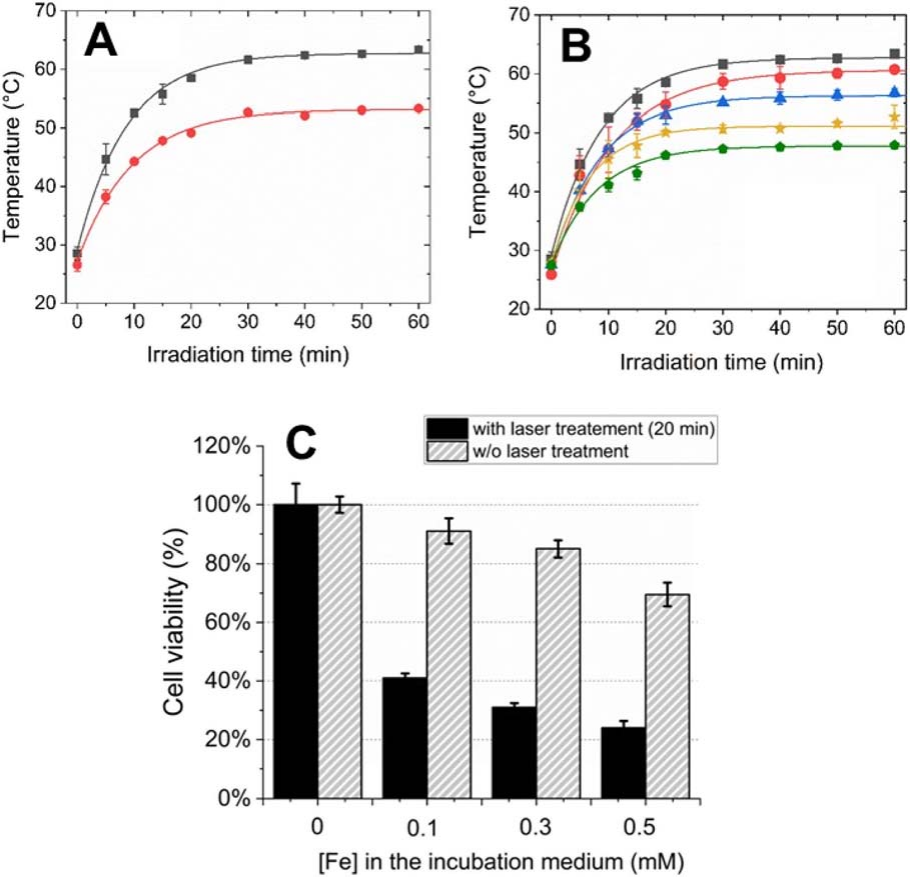
Assessment of the *in vitro* photothermal effect by measuring the temperature of the specimens at varying light irradiation times (maximum time: 1 h). (A) Comparison of temperature increase for PEG/Fe(iii)–GA (black) and PVP/Fe(iii)–GA (red) at the same iron concentration (5 mM). (B) Temperature increase for PEG/Fe(iii)–GA at varying iron concentrations: 5 mM (black), 3 mM (red), 1 mM (blue), 0.5 mM (yellow), and 0.1 mM (green). (C) Cell viability in the presence of PEG/Fe(iii)–GA at different concentrations, without (grey) and with laser treatment (black) for 20 minutes.

The photothermal effect of PEG/Fe(iii)–GA was evaluated *in vitro* by measuring the temperature increase of the specimens under varying light irradiation times (up to 1 hour), and compared to the reference PVP/Fe(iii)–GA at the same Fe(iii) concentration (5 mM). Fig. 7 shows the temperature enhancement upon laser light stimulation (*λ* = 680 nm, 0.48 W cm^−2^ irradiation density). As shown in Fig. 7A, PEG/Fe(iii)–GA induces a significant temperature increase upon light stimulation, with a rise of approximately 10 °C, much higher than the temperature increase observed for the PVP/Fe(iii)–GA control. The observed difference in photothermal efficacy between the two nanoparticles may be attributed to variations in particle size and the nature of the polymer coating. Furthermore, this discrepancy can be attributed to factors beyond mere absorption capacity, including the efficiency of light-to-thermal energy conversion and heat propagation within the material.^43^ The enhanced performance of PEGylated nanoparticles is likely due to a combination of these factors. It is important to note that after just 5 minutes of excitation, a temperature increase of 18 ± 2 °C was already observed. This rise continued, reaching 34 ± 3 °C at 30 minutes, and remained stable even when the excitation time was extended up to 1 hour. Notably, the temperature enhancement effect was also achieved at lower concentrations of PEG/Fe(iii)–GA, as shown in Fig. 7B. Interestingly, only about 15–20 minutes of irradiation were sufficient to nearly reach the maximum temperature increase. Furthermore, at the lowest tested concentration, a temperature of 45 °C was reached within 20 minutes, which could be sufficient to trigger cell death processes (Fig. S7†).^44^

Considering both the concentration and the irradiation time required to reach 45 °C, the PEG/Fe(iii)–GA nanoparticles developed in this study appear to be highly suitable for photothermal therapy (PTT) and show strong potential for cancer treatment applications. To assess their potential for PTT in cellular environments, TS/A cell viability was evaluated following incubation with PEG/Fe(iii)–GA at Fe(iii) concentrations ranging from 0 to 0.5 mM, followed by laser exposure for 20 minutes. A significant reduction in cell viability was observed, decreasing from 100% to 40% even at low nanoparticle concentrations upon light stimulation. Additionally, a progressive decline in cell viability was noted with increasing concentrations of PEG/Fe(iii)–GA (Fig. 7C).

**Fig. 8:**
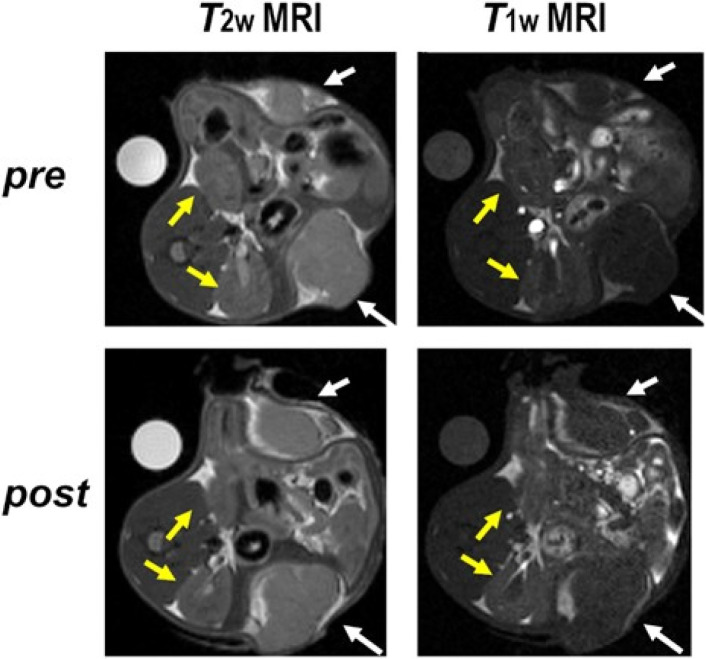
Representative *T*_2w_ and *T*_1w_ MR images pre and post (*t* = 15 min) i.p. administration of PEG/Fe(iii)–GA showing tumours (white arrows) and kidneys (yellow arrows).

Finally, preliminary proof-of-concept studies were conducted to assess the feasibility of using PEG/Fe(iii)–GA as an *in vivo* contrast agent through intraperitoneal (i.p.) injection in murine breast cancer models. The animal model was established by subcutaneously transplanting TS/A breast cancer cells. The dose of PEG/Fe(iii)–GA administered was selected to be as low as possible. MR images were acquired before and after the i.p. injection (up to 24 hours) to monitor the biodistribution of the nanoparticles. No toxic effects were observed in the mice following the i.p. injection. A slight *T*_1_ contrast was detected in the tumour region immediately after nanoparticle administration, with a signal enhancement of approximately 10% (Fig. 8 and S8†).

The observed contrast was lower than that typically achieved with intravenous (i.v.) injection of clinically approved Gd-based contrast agents (GBCAs) such as ProHance (Bracco Imaging S.p.A.). Several factors may contribute to this discrepancy. First, the dose of PEG/Fe(iii)–GA nanoparticles administered (0.05 mmol kg^−1^) was 12 times lower than the typical dose of GBCAs used in MRI (0.6 mmol per kg Gd for mice, corresponding to 0.1 mmol per kg Gd for human patients). Additionally, the absorption rate of nanoparticles *via* intraperitoneal injection is slower than that of i.v. administration. Lastly, the longitudinal relaxivity of PEG/Fe(iii)–GA at 7 T is lower than that of commercially available GBCAs. MRI analysis revealed that PEG/Fe(iii)–GA nanoparticles exhibit a predominantly hepatic slow elimination, with *T*_1_ signal enhancement observed in the liver and spleen, which remained relatively stable over the investigation period (*ca.* 30% and 5–10% enhancement for the liver and spleen, respectively, Fig. 8, S8 and S9†). No contrast enhancement was detected in the kidneys (Fig. 8 and S8†).

Considering these findings, the results demonstrate promising potential for future *in vivo* applications. However, further optimization of the nanoparticles is crucial, particularly in terms of selectivity for the target tumour and the administration protocol.

## Conclusions

A vast majority of metal-based nanoparticles and nanosystems designed as MRI diagnostic and theranostic probes contain Gd(iii) or Mn(ii) ions. This choice is primarily driven by the well-established ability of these ions to efficiently induce nuclear magnetic relaxation in nearby water protons. However, nanosized systems based on Fe(iii) ions offer a promising alternative, combining superior biocompatibility with an effectiveness nearly comparable to that of their Gd(iii)- and Mn(ii)-based counterparts.

We have developed nanoparticles composed of Fe(iii)-based coordination polymers and gallic acid, coated with a low-molecular-weight PEG shell. The paramagnetic ions are confined within the nanoparticles, limiting magnetic dipolar coupling to only the water molecules closest to the surface. The PEG shell was specifically chosen to enhance both the number and the average lifetime of the hydration layer, ultimately improving the relaxivity and overall effectiveness of the nanoprobes. This approach has proven highly effective, as the relaxivity values (per Fe(iii) ion) measured for PEG/Fe(iii)–GA nanoparticles are significantly higher than those of their PVP-coated counterparts (PVP/Fe(iii)–GA), exceeding a 150% increase at 3 T and 298 K. The chemical nature of the coating layer is therefore crucial, not only for isolating paramagnetic ions and preventing unwanted biotransformation but also for optimizing their interaction with the surrounding water molecules.

PEG/Fe(iii)–GA nanoparticles have also shown highly favorable properties for photothermal therapy (PTT), making them particularly promising for cancer treatment applications.

Additionally, their aqueous suspensions remain stable for weeks, exhibit a high biocompatibility profile, and provide a reasonable MRI contrast *in vivo* experiments, especially considering the low administered dose.

Although prior studies have explored similar Fe(iii) nanoparticles for biomedical applications, our research uniquely delivers the first comprehensive, quantitative analysis of their relaxation properties across a wide range of temperatures and frequencies. We emphasize that relaxivity, the key determinant of MRI contrast agent efficacy, is intrinsically frequency-dependent. Only through a detailed frequency-dependent investigation can we reveal the underlying mechanisms that govern their MRI contrast capabilities.^33,36^ Our study fills this critical knowledge gap by providing a rigorous, quantitative analysis of relaxometric profiles, systematically examining the influence of both applied magnetic field and temperature. This approach establishes the basis for the rational design of improved Fe(iii)-based nanoparticles. Moreover, we have shown that manipulating the nanoparticle coating does not diminish their photothermal performance; rather, it provides a valuable tool for fine-tuning and optimizing this property.

Although there is still room for further improvements and optimizations, these results strongly suggest that Fe(iii)-based nanosystems represent an effective and attractive option for the development of safer and more sustainable MRI probes.

## Author contributions

Conceptualization: MB, FC and GBG. Supervision MB, FC, GF. Funding acquisition: MB, GBG, FC and GF. MR and GBG carried out the synthesis and characterization of the nanoparticles. FC carried out the relaxometric study. GF, EDG and AC carried out the PT and MRI experiments. FC, MB and MR analysed the relaxometric data. FC, MC, GF and MB wrote the original draft. All the authors reviewed the manuscript.

## Conflicts of interest

There are no conflicts to declare.

## Supplementary Material

NA-007-D5NA00250H-s001

## Data Availability

The datasets supporting this article have been uploaded as part of the ESI.† Additional data can be provided upon reasonable request from the authors.
