# Supplementary material for: Comprehensive relaxometric analysis of Fe(iii) coordination polymer nanoparticles for T1-MRI: unravelling the impact of coating on contrast enhancement
Source: Nanoscale Adv. 2025 May 9;7(12):3792–802. doi: 10.1039/d5na00250h (PMC12082339; doi:10.1039/d5na00250h)
Supplement: NA-007-D5NA00250H-s001 [file NA-007-D5NA00250H-s001.pdf]

Supplementary Information (SI) for Nanoscale Advances.
This journal is © The Royal Society of Chemistry 2025

Supporting information for

Comprehensive Relaxometric Analysis of Fe(III) Coordination Polymer Nanoparticles for T_1-MRI: Unravelling the Impact of Coating on Contrast Enhancement

Marco Ricci,[a] Fabio Carniato,[a,b] Alessia Corrado,[c] Giuseppe Ferrauto,*[c] Enza Di Gregorio,[c] Giovanni Battista Giovenzana,[d,e] Mauro Botta*[a,b]

[a] *Dipartimento di Scienze e Innovazione Tecnologica Università del Piemonte Orientale, Viale T. Michel 11, Alessandria 15121, Italy.*
[b] *Magnetic Resonance Platform (PRISMA-UPO), Università del Piemonte Orientale, Viale Teresa Michel 11, 15121 Alessandria, Italy*
[c] *Department of Molecular Biotechnology and Health Sciences, University of Torino, 10126 Torino, Italy.*
[d] *Dipartimento di Scienze del Farmaco, Università del Piemonte Orientale, Largo Donegani 2/3, 28100 Novara, Italy.*
[e] *Magnetic Resonance Platform (PRISMA-UPO), Università del Piemonte Orientale, Via Bovio 6, 28100 Novara, Italy*

* mauro.botta@uniupo.it; giuseppe.ferrauto@unito.it

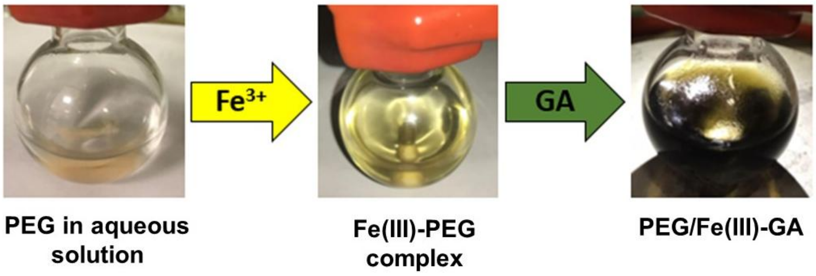

Figure S1. Digital photographs of solutions obtained during the synthesis of PEG/Fe(III)-GA nanoparticles.

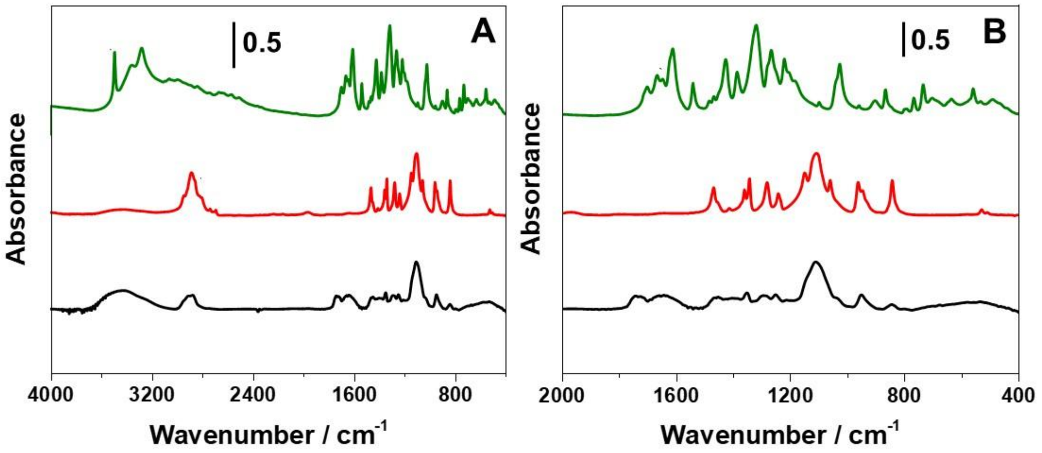

Figure S2. A) FT-IR spectra of gallic acid (green), PEG (red) and PEG/Fe(III)-GA (black); B) IR spectra in the 2000-400 cm^{-1} range.

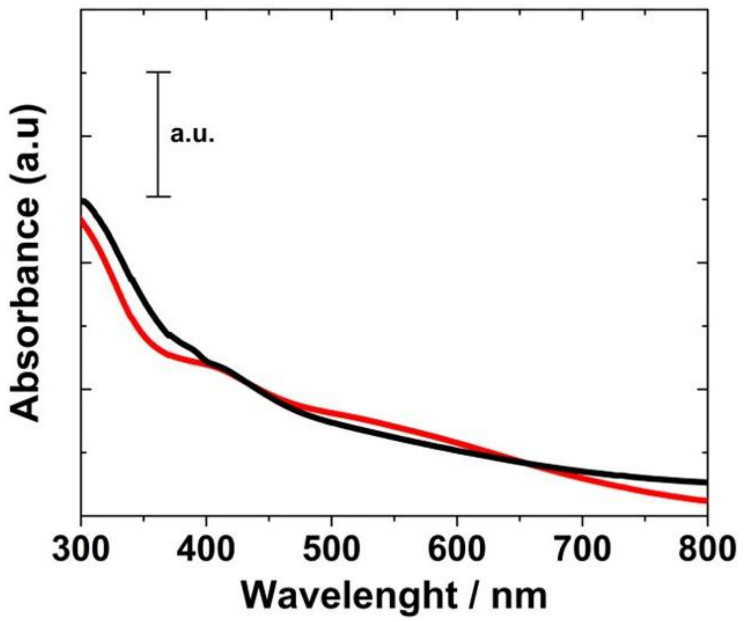

Figure S3. Comparison of the UV-Vis spectra of PEG/Fe(III)-GA (black) and PVP/Fe(III)-GA (red) at the same Fe(III) concentration (0.1 mM) and pH 7.4.

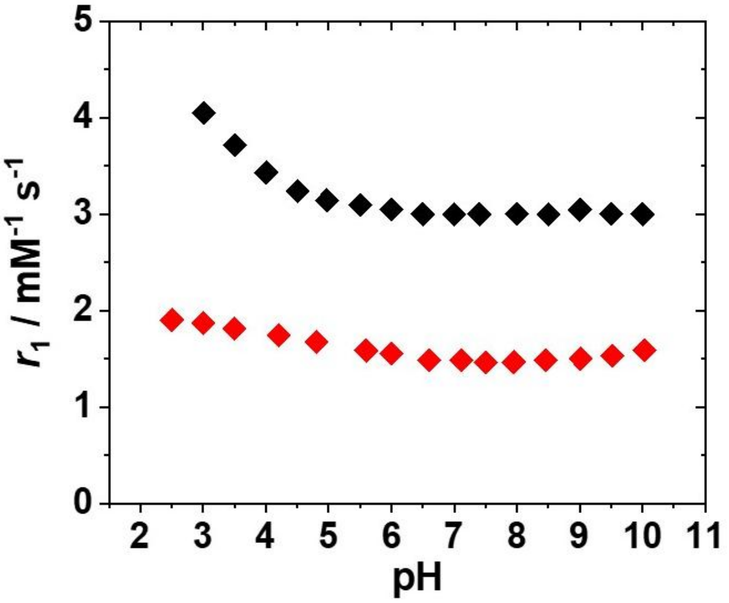

Figure S4. pH dependence of r_1 at 298 K and 32 MHz of PEG/Fe(III)-GA (black) and PVP/Fe(III)-GA (red).

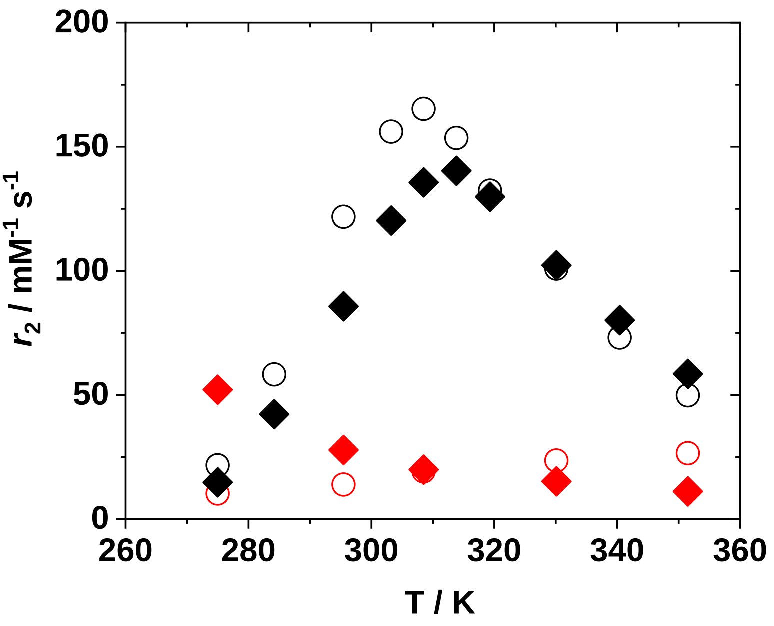

Figure S5. [17]O r_2 values of the suspensions at different pH values: ♦ = PEG/Fe(III)-GA, pH 7.4, ♦ = PEG/Fe(III)-GA, pH 3.0, ○ PVP/Fe(III)-GA, pH 7.4, ○ PVP/Fe(III)-GA, pH 3.0.

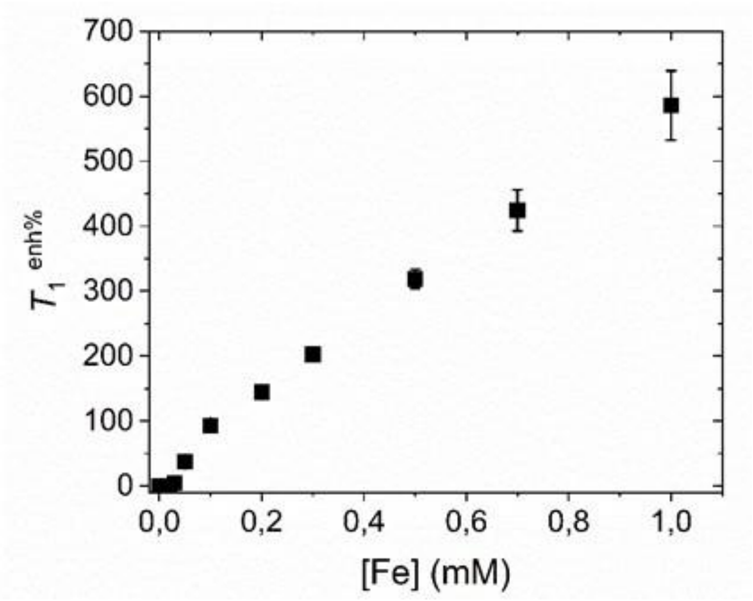

Figure S6. $T_1^{enh\%}$as a function of the Fe^{3+} concentration for PEG/Fe(III)-GA suspension at 7.1 T and 298 K.

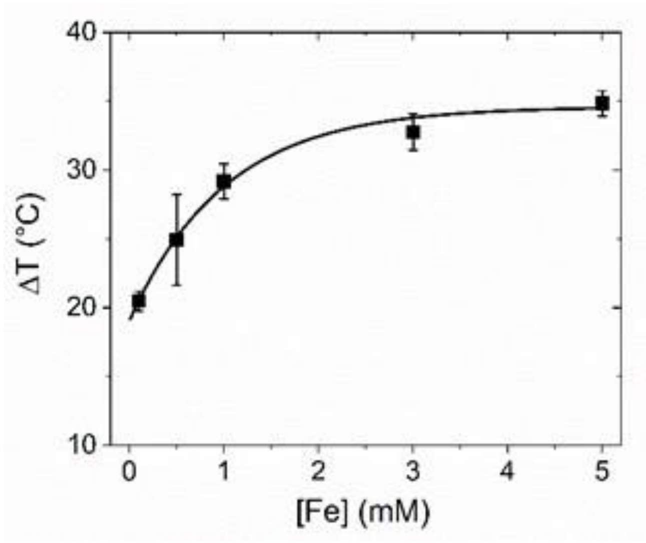

Figure S7. ΔT vs. [Fe^{3+}] for 1 h of irradiation for PEG/Fe(III)-GA suspension.

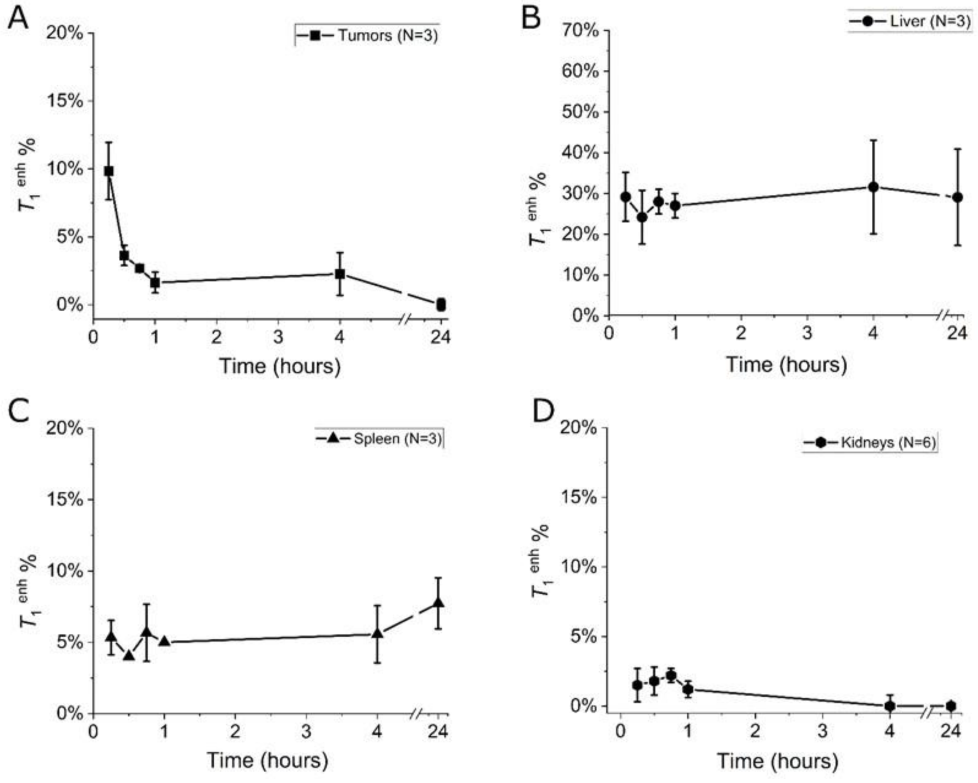

Figure S8. Signal Enhancement (%) in T_{1w}-MR images for tumors (A), liver (B), spleen (C) and kidneys (D) followed for 24h after i.p. administration of PEG/Fe(III)-GA.

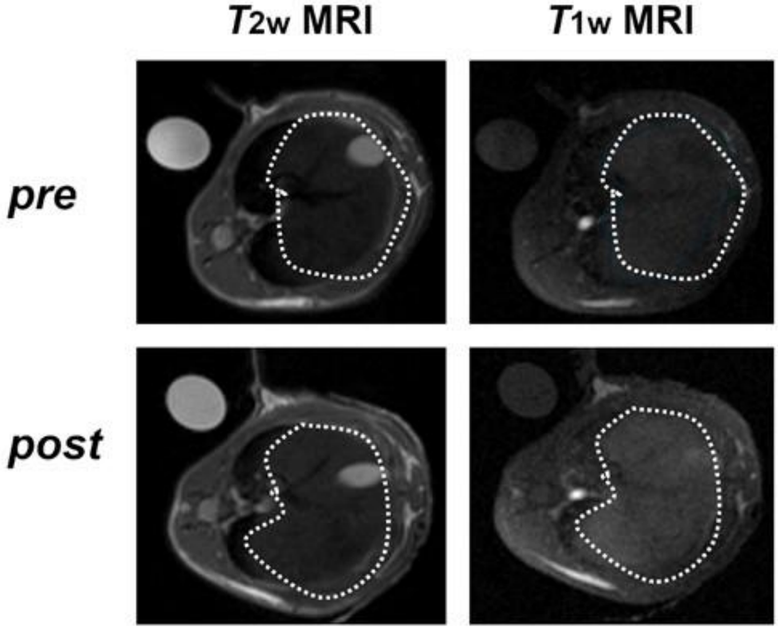

Figure S9. Representative T_{2w} and T_{1w} MR images pre and post (t = 15 min) i.p. administration of PEG/Fe(III)-GA showing liver (indicated by dotted ROI).
